# Control interno de la calidad – bases del pasado, situación presente y futuras tendencias

**DOI:** 10.1515/almed-2022-0028

**Published:** 2022-05-26

**Authors:** Carmen Ricós, Pilar Fernandez-Calle, Maria Carmen Perich, James O. Westgard

**Affiliations:** Sociedad Española de Medicina del Laboratorio (SEQC^ML^), Barcelona, España; Departamento de Medicina del Laboratorio, Hospital Universitario La Paz, Madrid, España; University of Wisconsin School of Public Health, Madison, WI, USA

**Keywords:** control de calidad interno, especificaciones de actuación, requerimientos clínicos

## Abstract

**Objetivos:**

Este artículo ofrece una síntesis de los modelos de control interno de la calidad analítica usados, desde mediados del siglo XX hasta los que están en vigor actualmente y pretende dar una proyección de cómo debería ser el futuro en esta materia concreta.

**Métodos:**

El material usado es la recopilación bibliográfica de los distintos modelos de CIC publicados. El método de estudio ha sido el análisis crítico de dichos modelos, debatiendo los pros y contras de cada uno.

**Resultados:**

Los primeros modelos se basaron en el análisis de materiales control y se fijaron como límites de aceptabilidad múltiplos de la desviación estándar del procedimiento analítico. Más adelante se sustituyeron estos límites por valores relacionados con el uso clínico de los exámenes del laboratorio, principalmente los derivados de la variación biológica. Para las pruebas sin material control estable se desarrollaron métodos basados en análisis replicados de especímenes de pacientes, que se han perfeccionado recientemente, así como la métrica sigma, que relaciona la calidad deseada con la prestación analítica para diseñar un protocolo de alta eficacia. La tendencia actual es matizar el control interno teniendo en cuenta la carga de trabajo y el impacto de un fallo analítico en la información sobre el paciente.

**Conclusiones:**

Se indican los puntos fuertes resaltados a la luz de esta revisión, los puntos débiles que todavía se emplean y deberían eliminarse, así como se da una proyección de futuro encaminada a promover la seguridad de los exámenes del laboratorio.

## Introducción

La medicina del laboratorio es una disciplina del área de la medicina que proporciona información del estado de salud de cada paciente e impacta sobre el mismo, aunque no se dispone de datos objetivos que cuantifiquen este impacto [1].

Por ello, el profesional del laboratorio debe asegurar la fiabilidad de sus informes, para evitar que los clínicos puedan tomar decisiones incorrectas que afecten a la salud del paciente.

El trabajo diario del laboratorio incluye actividades analíticas y extra-analíticas, la calidad de las cuales ha sido ampliamente estudiada [2], [3], [4], [5], [6], [7] y, en coherencia, la Sociedad Española de Medicina del Laboratorio (SEQC-ML) dedica tres grupos de trabajo para implementar y divulgar los conocimientos correspondientes: comisiones de calidad analítica y extra-analítica, de acreditación de laboratorios, así como comité de programas de garantía externa de la calidad www.seqcinternational.com/en/society/management-council/_id:7/.

La fase analítica del proceso global del laboratorio se centra en el examen de las magnitudes biológicas que constituyen los fluidos humanos. En este artículo una magnitud biológica de un determinado fluido humano investigada en el laboratorio se designa con el término “mensurando”.

Es indispensable implementar un sistema de aseguramiento de la calidad de los procedimientos analíticos utilizados en el laboratorio que sea robusto, que esté basado en la ejecución de estrategias de control interno de la calidad, así como en la participación en programas externos de garantía de la calidad, para producir resultados fiables (exactos y repetitivos). Otros elementos del sistema de gestión de la calidad, como monitorización de indicadores de la calidad, auditorías, etc. no forman parte de este artículo.

El objetivo del control interno de la calidad es monitorizar el proceso analítico para evitar producir información errónea sobre el estado de salud del paciente.

Este artículo ofrece una visión histórica, a través de una síntesis de los modelos de control interno de la calidad (CIC) analítica usados, desde mediados del siglo XX hasta los que están en vigor actualmente y pretende dar una proyección de cómo debería ser el futuro en esta materia concreta.

## Materiales y métodos

El material usado en este estudio es la recopilación bibliográfica de los distintos modelos de CIC utilizados.

El método de estudio ha sido el análisis crítico de dichos modelos, debatiendo los pros y contras de cada uno

## Resultados y discusión

### Bases del pasado

El modelo más antiguo, desarrollado en los años 50, estaba basado en un criterio estadístico a partir del cálculo de la media de un número de resultados del examen de un mensurando en la misma muestra de un material control. Se apoyaba en el uso de un gráfico bidireccional con los resultados control en el eje de abscisas y la media y desviación estándar en el eje de ordenadas. Los límites de aceptabilidad se definían como 2 desviaciones estándar por encima y debajo de la media, sobre la base de que si el 95% de los resultados entraban dentro de estos límites el procedimiento analítico se considera bajo control [8, 9]

En los años 80s James Westgard evaluó esta aproximación en el contexto de los sistemas analíticos automáticos, que permitían utilizar varios controles a niveles de concentración distintos (generalmente dentro y fuera del intervalo de referencia biológico) para cada mesurando. Se dio cuenta de que emplear los límites de control basados en ± 2 desviaciones estándar causaban un alto nivel de falsos rechazos. Se propusieron reglas control alternativas para limitar los falsos rechazos y al mismo tiempo maximizar la detección de errores mediante la aplicación de reglas múltiples. Las reglas control se identificaron con abreviaciones, tales como 1_3s_ que indica que se rechaza una serie cuando 1 resultado control excede los límites definidos por la media ± 3 desviaciones estándar. Un ejemplo de multi-regla fue 1_3s_/2_2s_/R_4s_/4_1s_/10_x_ [10, 11]. La primera regla fue seleccionada para mantener una baja probabilidad de falso rechazo (P_fr_) mientras que el efecto aditivo de las restantes reglas aumentaba probabilidad para detectar errores (P_ed_). Este algoritmo de multi-reglas fue implementado en muchos analizadores automáticos, permitiendo al profesional del laboratorio seleccionar la regla operativa a aplicar en su procedimiento analítico.

En aquel momento, las aplicaciones de control interno de la calidad estaban descritas propiamente como procesos estadísticos, que no tenían en cuenta el uso médico de las determinaciones del laboratorio. Los límites de control estaban basados en la variabilidad observada del procedimiento analítico, sin ninguna conexión con el uso clínico de la prueba. Este criterio tenía que ser utilizado en la evaluación del método, antes de su implementación en la rutina diaria. El concepto de “Error total admisible” (*Allowable Total Error*) fue entonces introducido, como la forma de expresar el requisito de la calidad en la evaluación y desarrollo de métodos analíticos [12].

El principal punto débil de este abordaje era que, al basarse en un criterio meramente estadístico, los márgenes de tolerancia estaban en consonancia con el rendimiento analítico inherente al método. Además, no era posible hacer una evaluación de la necesidad de mejora de las prestaciones de los distintos métodos analíticos puesto que no se podía conocer en qué grado los rendimientos analíticos de los diferentes métodos cubrían las necesidades clínicas.

Otro punto débil era que laboratorios con distintas prestaciones analíticas usaban distintos límites control, con lo que no se impulsaba la armonización.

Durante los años 80 y hasta mediados de los 90, el profesor sueco CH de Verdier y el profesor danés Mogens Horder realizaron una serie de estudios para definir los requisitos de la calidad y relacionarlos con el control de la calidad, bajo los auspicios de NORDKEM, Nordic Clinical Chemistry Projects, que incluían a todos los países escandinavos [13], [14], [15], [16].

Ya en la segunda mitad de los 90, un grupo plurinacional de expertos, bajo el auspicio del *Standards Measurement and Testing Programme* propuso una serie de recomendaciones para el control interno de la calidad analítica que se resumían en [17].

– El control interno debería estar integrado en el sistema de gestión de la calidad del laboratorio.
– Debería combinarse con un programa de control externo que facilite la trazabilidad de las determinaciones a patrones internacionales.
– Debería asegurar que se cumplan unas especificaciones de la calidad relacionadas con la variación biológica, cuando sea posible.
– Para un procedimiento analítico con frecuencia de error alta, el protocolo de control interno debería tener máxima P_de_ con la mínima P_fr_ posible, para estimular un buen sistema de resolución y prevención de problemas. La tendencia general debería ser a poder usar reglas operativas más relajadas, pero manteniendo siempre una P_fr_ baja.
– Los resultados del paciente para todas las magnitudes biológicas solicitadas deben tenerse en cuenta antes de entregar el informe, pero nunca sustituyen a las determinaciones control.
– Es mejor prevenir los errores que tenerlos que corregir.

Las recomendaciones de Hyltoft y cols [17]. Introdujeron el concepto de especificación de la calidad basada en la biología y en el uso clínico de la prueba y no en la estadística, como se había hecho anteriormente. Esta aproximación tenía en cuenta ya el uso final de las determinaciones del laboratorio médico.

En la conferencia internacional de consenso de Estocolmo en 1999 se estableció una estrategia jerárquica de especificaciones de la calidad analítica, de manera que desde ese momento los límites de tolerancia pasaron a calcularse para cumplir esas especificaciones [18]. En esta conferencia se presentó una base de datos de estimados de variación biológica, realizada mediante la recopilación de los artículos existentes hasta la fecha por la Comisión de Calidad Analítica de la SEQC, en la que se incluía el cálculo de las especificaciones de calidad [19]. Esta base de datos se actualizó cada 2 años hasta el 2014 y se expandió universalmente gracias a su inclusión en la web de Westgard [20].

Quizás el punto débil de este otro tipo de abordaje residía en la naturaleza del material control (estabilización, adición de aditivos, etc.), de manera que eran no conmutables y por tanto podían comportarse de distinta manera que las muestras de pacientes, frente a los distintos métodos analíticos disponibles. Algunas organizaciones utilizaban muestras control de procedencia humana congeladas a −80 °C, distribuidas en alícuotas sin ninguna otra manipulación, que eliminaban la limitación mencionada [21]. El problema en este caso solía ser la dificultad de conseguir cantidad suficiente de material control, además del coste de la infraestructura necesaria para su mantenimiento (congelación a −80 °C).

Para aquellos mensurados en los que no se disponía de material control estable se propuso en 1960 calcular el desplazamiento del promedio de los resultados las muestras de pacientes (Control con medias móviles) (*Moving Average*) como identificador de desviación analítica [22, 23]. La condición necesaria para aplicar este modelo era que se tratara de mesurados con población estable, por ejemplo, índices hematimétricos, pero no para glucosa o creatinina en situación de emergencia, por ejemplo.

Además, si se analizaban por duplicado algunas de dichas muestras y se calculaba la desviación estándar de las diferencias entre duplicados, se obtenía también un estimado de la imprecisión analítica. Cembroswki y cols. propusieron en 1984 unas guías de práctica clínica para implementar este modelo [24].

### Situación presente

Entrado ya el siglo XXI se desarrollaron modelos de CIC basados en el rendimiento del procedimiento analítico mediante la métrica Sigma, que es la relación entre la especificación de la calidad analítica que se desea obtener, expresada en términos de error total analítico admisible (ET_a_, %) y la prestación del procedimiento analítico, expresada en términos de error sistemático en valor absoluto (absES,%) e imprecisión (CV,%).

$$\text{Sigma}=\left({\text{ET}}_{\text{a}}-\text{absES}\right)/\text{CV}$$

Cuanto mayor es el valor sigma, más relajado puede ser el procedimiento de CIC y viceversa [25].

Si el control de la calidad se centra en un único proceso analítico, el término ES podría considerarse cero, porque lo que se pretende es detectar cambios en el proceso que podrían implicar error médico. Sin embargo, cuando se controla más de un proceso (p.e. dos sistemas analíticos usados indistintamente en el mismo o en distintos laboratorios de un centro sanitario), sí hay que considerar el ES [26]. Además, diferentes lotes de calibrador y diferentes lotes de reactivo pueden producir ES de repercusión considerable e incluso clínicamente significativos de forma que puede llevar a error en la monitorización de los pacientes (p.e. PSA tras radioterapia como tratamiento de erradicación del carcinoma de próstata).

A continuación, se describen los tres puntos que se consideran claves en un protocolo de control interno.

#### Punto clave 1. Especificaciones de la prestación analítica

El error total tolerable debería estar basado en una de las tres propuestas de la 1^a^ Conferencia Estratégica IFCC de Milán [27]. Idealmente la especificación para la prestación analítica debería basarse en el impacto del error analítico sobre la información del estado de salud del paciente; sin embargo, es muy difícil establecer la relación directa entre esos dos factores [26].

Esta es la razón por la cual la variación biológica es la opción más ampliamente utilizada para establecer las especificaciones de la prestación analítica, porque asegura el uso correcto de los resultados del laboratorio. Para los mesurados sin datos conocidos de variación biológica o muy débilmente regulados fisiológicamente, el estado del arte entendido como el más alto nivel de prestación analítica técnicamente posible es otra opción aceptada en la conferencia de Millán [27, 28].

Los datos de variación biológica están actualmente disponibles en la página web de la EFLM [29]. Esta nueva base de datos ha sido elaborada a partir de una revisión exhaustiva de los artículos incluidos en la primera base de datos [19, 20] y de la aplicación de una estrategia de búsqueda eficaz, de forma que se recopilara el mayor número posible de artículos sobre variación biológica. El *Working Group* de Variación Biológica de la EFLM desarrolló una herramienta de evaluación (*Critical appraisal checklist BIVAC*) con unos criterios de evaluación de la calidad de la metodología de los estudios sobre variación biológica muy rigurosos que, al aplicar posteriormente un meta-análisis, mejoran y aseguran la robustez y fiabilidad de los estimados de variación biológica obtenidos [30].

El estado del arte puede extraerse del programa de garantía externa de la calidad en que participe el laboratorio (percentil 20 o 30 de las desviaciones obtenidas por los participantes) [31]. La evaluación anual de los programas de la SEQC muestra los percentiles 20, 30, 50, 70 y 90 de cada mensurando (https://www.contcal.org/qcweb/Qcw_Documentacio.aspx)

#### Punto clave 2. Prestación del procedimiento analítico

La prestación del procedimiento analítico se mide en el laboratorio individual, en condiciones de rutina, en términos de coeficiente de variación (CV) como medida de la imprecisión y de desviación porcentual respecto al valor diana (VD) del control como medida del sesgo. Si se usa el material control suministrado por el proveedor del sistema analítico, se recomienda calcular el CV y el VD del material control con un mínimo de 10 determinaciones a lo largo de 10 días consecutivos, en condiciones estables del procedimiento analítico (mismos reactivos, mismo operador técnico, etc.) y actualizarlo si es necesario después de observar un período más largo (6–12 meses) [26]. Resulta muy práctico pactar con el proveedor un mismo lote de material control por un largo período.

En cuanto al material control, lo recomendable es usar controles estabilizados independientes del fabricante del sistema analítico (control de tercera-parte*, third-party control)* para comprobar que el procedimiento analítico no se desvíe respecto al valor diana del material control; la ISO 15189 [32] lo indica específicamente. Se puede considerar como valor diana el promedio de resultados obtenidos por el grupo par de laboratorios, esto es, usuarios del mismo lote de material control, con el mismo sistema analítico (método, instrumento). De esta forma la media con la que se realiza la comparación y se cuantifica la desviación del laboratorio individual respecto al grupo par, es estadísticamente robusta. Permite también evaluar la armonización existente entre laboratorios, aunque no la estandarización entre ellos porque no hay trazabilidad a patrones de referencia [33, 34].

Indirectamente este material puede dar una visión general de su desviación respecto a los otros participantes, si el laboratorio tiene acceso a los resultados de usuarios de otros proveedores, igual que en un programa externo. Existe la tendencia de denominar este tipo de control como control interno/externo, pero es una terminología equívoca, ya que, aunque este tipo de control proporciona elementos de comparación con otros laboratorios, las muestras no se analizan de forma ciega, que es uno de los requisitos principales de un programa de control externo. Por tanto, este tipo de control ha de utilizarse como CIC y complementarse con la participación en un programa de garantía externa de la calidad EQA.

Debido a que el CIC debe asegurar no solo la reproductibilidad del procedimiento analítico, sino también que no se desvíe respecto al valor diana, algunos autores insisten en incorporar el concepto de trazabilidad a patrones mediante el uso de controles conmutables con las muestras de los pacientes; es decir, usar controles conmutables y con valores asignados por métodos de referencia certificados [35, 36]. Sin embargo, este modelo puede resultar muy difícil de llevar a la práctica, por la escasa disponibilidad de controles conmutables para el uso diario, como ya se ha mencionado anteriormente.

Ante la dificultad de utilizar controles conmutables en la rutina diaria, sería importante al menos, participar en un programa de evaluación externa de la calidad que utilice este tipo de material control conmutable, y a ser posible con valor asignado por método de referencia. Al ser un control discontinuo, es logística y económicamente asequible. Estos materiales control se pueden considerar como patrones de referencia y permiten evaluar la exactitud de las determinaciones analíticas [37, 38] y, por extensión, la estandarización entre laboratorios.

#### Punto clave 3. Control interno de la calidad basado en los resultados de los pacientes

Una alternativa para obviar el problema de la conmutabilidad de los materiales control es complementar el sistema de CIC con la estrategia de utilizar los resultados de los pacientes como un CIC adicional.

Ya se ha mencionado anteriormente el modelo de control con medias móviles [22, 23]. En los últimos años se ha desarrollado mucho, aprovechando los sistemas informáticos actuales [39, 40]. La IFCC ha publicado recomendaciones para verificar y validar un protocolo de control con medias móviles, antes de su implementación en el laboratorio [41].

Para aumentar el potencial de detección de error de este modelo, es importante acotar la población de pacientes que entra en el cálculo de la media; para ello, se realiza un análisis de regresión multivariante previo al cálculo, que incluye la media de 2000 resultados de pacientes y otras variables independientes como edad, sexo, pacientes ingresados vs ambulatorios, así como diagnóstico y planta de ingreso [42, 43].

La Comisión de Calidad Analítica de la SEQC ha impartido un curso de formación continuada en enero 2022, donde se realiza una revisión de esta estrategia y se pone énfasis en los factores a optimizar en la implantación de este modelo [44]. Estos factores son:

– Límites de truncado para seleccionar resultados de pacientes a promediar,
– Algoritmo de cálculo utilizado (media, mediana, media ponderada).
– Límites de control, que han de estar en función de las especificaciones de la calidad que el laboratorio se ha planteado.

Un artículo reciente de Bayat y cols [45] compara el modelo clásico de CIC basado en materiales control y multi-reglas con un modelo basado en los resultados de los pacientes. Se dan cuenta que el método clásico es muy práctico en procedimientos analíticos con sigma alta (sigma>4) (más simple y rápido para liberar los resultados), mientras que la multi-regla acoplada a unas medias móviles puede aumentar la detección de errores en procedimientos con baja sigma (sigma<4), a pesar de su complejidad.

El punto débil de esta aproximación es que con ella no es posible medir directamente el error sistemático, puesto que no se conoce el verdadero resultado de las muestras de pacientes, pero sí que detecta un cambio del mismo. Este problema se obviaría combinando el CIC de medias móviles con un control externo adecuado para estimar el error sistemático del laboratorio.

Se pueden realizar análisis duplicados de algunas muestras de pacientes a diario, calcular para cada mensurando las diferencias entre duplicados y obtener el parámetro denominado *Delta de pacientes* (*Patient Delta).* El promedio de los Delta de pacientes (*Average of Delta, AoD*) es idealmente cero y si se desplaza, indica un cambio del error sistemático del procedimiento analítico [46]. Cervinski y cols. han aplicado este modelo al SIL del laboratorio para pacientes ingresados y han buscado el número de pares de muestras de pacientes óptimo para conseguir máxima detección del error sistemático [42].

El error aleatorio o imprecisión, expresada en términos de desviación estándar (ES), se puede calcular a partir de las diferencias entre los duplicados de pacientes, mediante la fórmula

$$\text{ES}={\left(\sum {\left(x_{2}-x_{1}\right)}^{2}/2n\right)}^{1/2}$$

Siendo *x*_2_ y *x*_1_ el segundo y primer resultado de cada par y *n* el número de pares de muestras de pacientes analizados.

Un excelente ejemplo de CIC de amplio enfoque sobre la base de resultados de pacientes, es la iniciativa a gran escala del proyecto Empower, liderado por Linda Thienpont y cols [47]. Este proyecto recoge los resultados de pacientes de diferentes laboratorios, con un registro del instrumento utilizado, método, lote de calibrador y lote de reactivo. Con ello se obtienen estadísticas muy potentes que proporcionan a fabricantes y laboratorios una visión realista de la calidad y comparabilidad, así como de la estabilidad de la prestación o de los motivos de aumento de variabilidad de los laboratorios participantes

A partir del año 2011 se integra el concepto de control de la calidad basado en la gestión del riesgo para el paciente dentro del CIC. El *Clinical and Laboratory Standards Institute* (CLSI) (indica que se deben considerar tres factores de riesgo) [48, 49]. Estos son:

– Frecuencia de ocurrencia de error analítico.
– Capacidad del modelo de CIC implantado para detectar un error.
– Severidad que el error pueda producir en el conocimiento del estado de salud del paciente.

Por ello, el protocolo de CIC debe prefijar la frecuencia en la que hay que pasar un material control y el número de niveles de concentración control. Esto lo hace en función del número de muestras de pacientes que atiende el laboratorio, de los niveles de control disponibles, de la imprecisión conocida del método y del riesgo que se quiera asumir a tenor de la repercusión en los pacientes (no es lo mismo asumir un 10% de pacientes mal clasificados para una magnitud como la troponina que diagnostica infarto de miocardio que para los triglicéridos o la ALT). Es decir, hay que revisar la frecuencia con que debe procesarse el CIC para evitar entregar informes de resultados de pacientes obtenidos en series analíticas incorrectas [50], [51], [52]. Para ello existen programas informáticos que pueden ayudar a los laboratorios en el manejo de estas estrategias.

**Figura 1:**
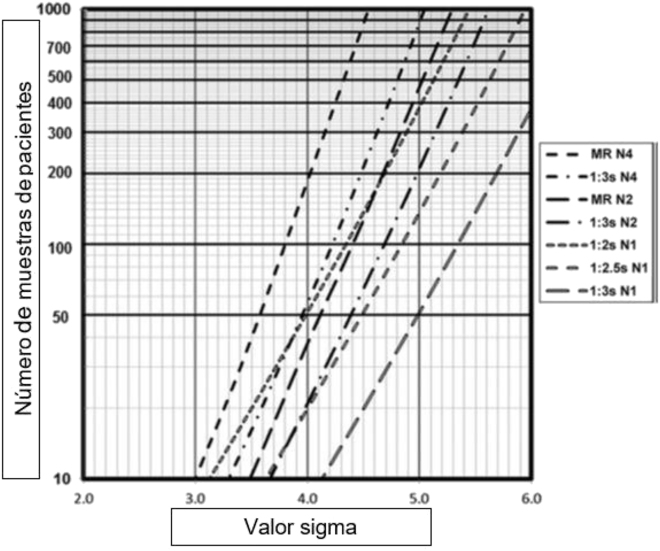
Nomograma basado en métrica σ y carga de trabajo para seleccionar la regla operativa de CIC. Obtenido de: Westgard JO et al. Clin Chem 2018;64:259–296 [52]. MR N4: multi-regla con 4 controles por serie. Es la regla 1_3s_/2_2s_/R_4s_/4_1s_ MR N2: multi-regla con 2 controles por serie. Es la regla 1_3s_/2_2s_/R_4s_ N: resultados control.

**Figura 2:**
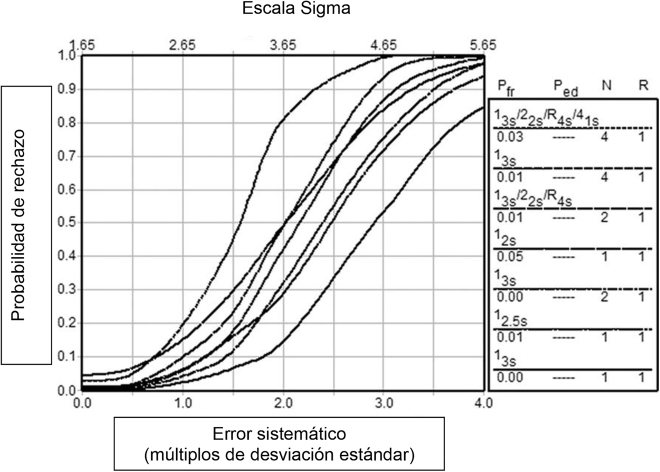
Curvas de potencia de las reglas operativas de CIC incluidas en el nomograma. Obtenido de Westgard JO et al. Clin Chem 2018;64:259–296 [52]. P_fr_: probabilidad de falso rechazo P_ed_: probabilidad de detección de error N: número de determinaciones control R: número de series analíticas en las que se aplica la regla operativa de CIC.

Si no se dispone de un programa informático de soporte, resulta muy práctico utilizar el nomograma propuesto por Westgard y cols (Figura 1), donde a partir del valor sigma y del número de muestras de pacientes a analizar (carga de trabajo) se elige una o varias reglas operativas de control de la calidad; éstas se pueden llevar a un gráfico de curvas de potencia, para visibilizar su probabilidad de detección de error (P_de_) y de falso rechazo (P_fr_) (Figura 2) [52].

Este modelo facilita utilizar varias reglas operativas durante la jornada de trabajo: más estrictas en momentos críticos (p.e. calibración o ajuste del instrumento, cambio de reactivo, etc.) con máxima P_de_, y una regla más relajada para monitorizar las series analíticas durante el resto de la jornada con mínima P_fr_.

El punto fuerte del modelo clásico de las multi-reglas, ampliado con la estimación de la frecuencia del procesamiento de muestras control, es que resulta relativamente fácil de aplicar con las herramientas actuales: las multi-reglas están incorporadas en los analizadores automáticos y el nomograma mencionado en el párrafo anterior está publicado [52].

### Consideraciones sobre el POCT

Las pruebas realizadas a la cabecera del paciente (*Point of Care Testing*, POCT) merecen particular atención. Si producen resultados cualitativos o semi-cuantitativos, p.e prueba de embarazo o pruebas realizadas por el paciente, simplemente se analiza un control, positivo o negativo y el resultado debe ser acorde al control.

Si producen resultados cuantitativos en instrumentos de moderada complejidad (cartuchos o tiras reactivas), p.e HbA1C, glucosa en sangre o de alta complejidad, p.e. gases en sangre, es necesario procesar muestras control de la misma forma como se haría en el laboratorio central [26]; así mismo, se debería verificar la intercambiabilidad de los resultados de POCT con los del laboratorio central, como indica la norma ISO 22870 [53].También se pueden procesar muestras de pacientes en paralelo con el laboratorio central, si la localización del POCT lo hace posible. Venner y cols recomiendan un mínimo de 10 pacientes positivos y 10 negativos en la verificación inicial del instrumento de medida y 5 pacientes de cada tipo en la monitorización rutinaria del instrumento [54].

Se han desarrollado indicadores de la prestación para los POCT, que incluyen aspectos extra-analíticos, p-e. porcentaje de pruebas realizadas en relación a pruebas compradas, porcentaje de pruebas realizadas en laboratorio central con respecto a pruebas realizadas en laboratorio central y POCT, porcentaje de operadores no identificados. Para la fase analítica los indicadores suelen ser del tipo porcentaje de pruebas con CV dentro de la misma especificación que la aceptada para el laboratorio central, porcentaje de pruebas informadas en relación a pruebas realizadas [55].

Para evaluar la competencia técnica y las habilidades de los operadores, un indicador puede ser el porcentaje de pruebas realizadas por el operador de POCT con la mayor actividad en a cada área clínica en relación a todas las realizadas en dicha área.

Cuando existe relación con el laboratorio central, resulta muy importante implementar instrumentos que permitan el control remoto desde el laboratorio.

### Visión global actual

En 2017 y 2021 Sten Westgard ha elaborado dos encuestas a unos 700 laboratorios ubicados en los cinco continentes sobre CIC. Las preguntas realizadas se centran en dos aspectos: el modelo de CIC utilizado y la gestión inmediata realizada sobre la base de los resultados control [56, 57]. En la Tabla 1 se desgranan los principales resultados obtenidos en las dos encuestas.

**Tabla 1:** Encuestas mundiales sobre el modelo de CIC utilizado. Respuestas en % de laboratorios.

Modelo de CIC	2017	2021
Límites control		
DE del laboratorio	63	58
Insert fabricante del control	43	57
DE del grupo par	20	24

Regla de control		

1_2s_	55	59
Multi-reglas a todas las magnitudes	–	23
Multi-reglas a algunas las magnitudes	–	64

Muestras control-procedencia		

Fabricante del instrumento	64	67
Tercera-parte, líquido, valorado	44	43
Tercera-parte liofilizado	35	31
Tercera-parte, líquido, no valorado	30	20
Medias móviles	11	14

Frecuencia		

1 vez al día	49	54
Varias veces al día	41	46
Criterio del facultativo	38	38
Cálculo del riesgo para el paciente	14	NP
Al principio y al final de la jornada	13	NP
Por grupos de pacientes (p.e. cada 100)	9	NP

DE, desviación estándar; NP, no preguntado.

El modelo de CIC utilizado produce la siguiente información:

– El material control más empleado es el del fabricante del instrumento utilizado, mientras que se considera más adecuado emplear control fabricado por terceras partes, tal como recomienda la norma de acreditación de laboratorios clínicos ISO 15189 [32].
– Los límites control más usados son los marcados por la desviación estándar del propio procedimiento analítico, lo que solo es adecuado si se ha comprobado previamente que cumple la especificación para precisión aceptada por el laboratorio.
– La regla de control más empleada es, todavía, la 1_2s_, que generalmente tiene alta probabilidad de falso rechazo, por lo que se considera ineficiente.
– No parece que muchos laboratorios ajusten la frecuencia del control a su carga de trabajo, ni a la gestión del riesgo para el paciente, aspecto que debería tenerse en cuenta en el futuro inmediato. Sí que hacen caso de la norma ISO 15189 o de la legislación existente en el país encuestado (45%), que es un aspecto muy positivo Tabla 2.

**Tabla 2:** Encuestas mundiales sobre la gestión inmediata realizada por el laboratorio. Respuestas en % de laboratorios.

Gestión inmediata	2017	2021
Procedimiento fuera de control		
Buscar causas antes que repetir muestras de pacientes	78	79
Repetir muestra control	78	68
Preparar nueva muestra control	64	55
Re-calibrar el instrumento	16	20
Avisar inmediatamente al fabricante	2	4

Repetición de muestras de pacientes		

Grupos determinados	33	31
Todos los del día	32	33
Solo con resultados anormales	20	24
Solo con resultados cercanos al control fallado	13	14

Entregar resultados de pacientes cuando falló el control		

Nunca	54	48
Pocas veces (<10/mes)	30	30

La gestión inmediata tras un aviso de procedimiento fuera de control trasluce la siguiente situación:

– Se observa una tendencia a buscar las causas del fallo antes de repetir la determinación control o preparar nueva muestra control, lo cual es muy positivo. Afortunadamente, muy pocos laboratorios se limitan a avisar al fabricante o distribuidor del sistema analítico, que es la opción menos adecuada.
– La repetición de muestras de pacientes seleccionadas (cercanas a la determinación del control que falló o con resultados anormales) es la opción más usada y también la más adecuada. Nos parece poco razonable repetir todas las muestras de pacientes de la serie analítica, a no ser que fallara más de un resultado control; todavía hay un 30% de laboratorios anclados en esta usanza poco recomendable.
– Sorprendentemente, la mitad de los laboratorios encuestados entrega resultados de pacientes cuando falló el control, práctica totalmente desaconsejable.

En España se realizó una encuesta en el año 2015 sobre las especificaciones de la prestación analítica utilizadas en aquel momento [58]. La encuesta reveló que el 47% de los participantes seguía las especificaciones derivadas de la variación biológica, el 33% del estado del arte obtenido del programa externo o del consenso español sobre especificaciones mínimas [59], el 5% de las opiniones de clínicos, el 3% usaba las reguladas en otros países como CLIA [60] y Rilibäck [61], y el resto otros criterios.

Por otra parte, es imprescindible que los laboratorios implementen también una gestión a largo plazo de los datos aportados por el CIC, consistente en evaluar periódicamente (p.e. cada mes) la prestación y compararla con las especificaciones que el laboratorio considera aceptables. Si no se cumplen, debe revisar si ha sido consecuente con el protocolo diario de CIC (ha aceptado series con resultados control rechazables), si el mantenimiento de los sistemas analíticos es adecuado, si el personal técnico está bien formado, etc. [62, 63].

Como concluye Sten Westgard en su encuesta de 2021, los laboratorios deberían cambiar y adoptar mejores protocolos de CIC; de otro modo ponen en riesgo su viabilidad o, peor todavía, ponen en riesgo a los pacientes debido a informes analíticos de poca calidad.

## Conclusiones

Los puntos fuertes resaltados a la luz de esta revisión son:

– Existen protocolos de CIC bien establecidos para monitorizar la prestación analítica y detectar cambios en el error sistemático, usando resultados de muestras control y también de pacientes.
– Existe material control no conmutable fabricado por terceras partes y con información sobre otros laboratorios usuarios del mismo control.
– Los datos de VB para establecer los límites de control son altamente fiables y están disponibles para un elevado número de mensurados.
– La carga de trabajo y el impacto de un fallo analítico sobre el conocimiento del estado de salud del paciente es fácil de conocer y debe aplicarse en el protocolo de CIC.

Los puntos débiles que deben eliminarse son:

– Usar la regla de control 1_2s_ por elevada P_fr_.
– Usar límites de control basados en criterios únicamente estadísticos.
– Repetir determinaciones control sin buscar primero la causa del fallo.
– Repetir el análisis de todas las muestras de pacientes, sin investigar donde se produjo el fallo.
– Entregar resultados de pacientes cuando falló el control.

La proyección de futuro recomendada es:

– Usar controles de terceras partes.
– Desarrollar CIC con resultados de pacientes cuando no existe control estable.
– Usar especificaciones basadas en la VB.
– Potenciar que los organizadores de Control Externo y materiales de control interno puedan preparar controles conmutables y disponer de medios para almacenarlos en el laboratorio (−80 °C).
